# A longitudinal analysis of the economic cost of all phases of TB care in a low incidence setting

**DOI:** 10.5588/ijtldopen.25.0076

**Published:** 2025-07-09

**Authors:** L.C. Ramsay, S.K. Brode, E. Rea, K. Barrett, A. Hernandez, N. Iragorri, K. Liu, L. Macdonald, B. Sander

**Affiliations:** ^1^ University of Toronto, Toronto, ON, Canada;; ^2^ University Health Network, Toronto, ON, Canada;; ^3^ ICES, Toronto, ON, Canada;; ^4^ Toronto Public Health, Toronto, ON, Canada;; ^5^ Public Health Ontario, Toronto, ON, Canada.

**Keywords:** tuberculosis, Canada, cost of illness analysis, post-TB, PTLD

## Abstract

**BACKGROUND:**

Our objective was to estimate the attributable health care costs associated with TB in Ontario, Canada.

**METHODS:**

We conducted an incidence-based matched cohort study among individuals diagnosed with TB between April 1, 2002 to December 31, 2016. We matched exposed individuals 1:2 to unexposed individuals using hard and propensity score matching. Using phase-of-care costing, we calculated the mean attributable costs of TB, standardized to 10-day cost, for seven phases of illness: pre-diagnosis, initial treatment, continuation phase, remainder year 1, year 2, post-TB, and prior-to-death. We estimated survival-adjusted attributable mean 1-, 2-, and 5-year costs.

**RESULTS:**

We matched 6,456 individuals with TB to 12,443 individuals without TB. Mean (95% CI) attributable 10-day costs was highest in the pre-death phase at $2,656 ($2,207, $3,104), followed by the initial treatment phase at $1,693 ($1,608, $1,778). Hospitalization costs were the largest cost component in each phase. The mean attributable 1-, 2-, and 5-year survival-adjusted costs were $25,586, $30,178, and $33,370, respectively.

**CONCLUSION:**

Individuals with TB have higher health care costs over their lifetime (from pre-diagnosis until death) than individuals without TB.

TB remains a significant public health concern in Canada.^1–3^ In Ontario, Canada’s most populous province, over 600 individuals were diagnosed with TB annually since 2015.^4^ TB has a greater incidence among individuals born outside of Canada with 12.3 per 100,000 population compared to 0.3 per 100,000 population for non-Indigenous people born in Canada.^2^ While TB is usually curable, despite successful treatment, long-term sequelae are common, such as chronic respiratory conditions, resulting in lifelong health care needs.^5–8^ Without a clear understanding of these long-term costs, the economic burden of TB may be underestimated. Prior research estimated costs associated with TB infection and disease at 3 Canadian treatment centres using a chart review, identifying hospitalization and drug resistance as key cost drivers.^9^ Another study assessed health care resource use (not costs) using a matched cohort study to estimate the 5-year resource use among foreign-born individuals treated for respiratory TB in British Columbia, showing sustained increased resource use over time.^10^ However, these studies focus on specific populations or time frames, and there remains an important gap in understanding the long-term, population-based costs of TB disease in Canada. International costing studies have also estimated the costs of TB, focusing on aspects like hospitalization or drug therapy, while some include broader evaluations such as productivity losses.^11–15^ However, variability in methodologies and differences in health systems and demographics limit their applicability to the Canadian context.

We aim to estimate the short- and long-term health care costs of TB using a population-based cohort of individuals diagnosed with TB in Ontario, Canada.

## METHODS

We report our study following the RECORD statement for observational studies (Supplementary Data Table S1).^16^ This project was approved by the Research Ethics Board at the University Health Network and the University of Toronto.

### Study design, setting, and participants

We conducted an incidence-based matched cohort study to examine the attributable costs associated with TB from the health care payer perspective in Ontario, Canada. Ontario has a population of nearly 16 million people, approximately 40% of Canada’s total population.^17^ We used population-based health administrative data housed at ICES. ICES is an independent, non-profit research institute whose legal status under Ontario’s health information privacy law allows it to collect and analyze health care and demographic data, without consent, for health system evaluation and improvement. Datasets include information about publicly funded health care encounters and population demographics. In addition, integrated Public Health Information System (iPHIS) and Public Health Ontario Laboratory (PHOL) data were brought into ICES (Supplementary Data Table S2). All datasets were linked using unique encoded identifiers and analyzed at ICES.^18^

### Exposed individuals (with TB)

We identified individuals as exposed, i.e., people with active TB disease, based on diagnosis in Ontario (provincial TB case definitions available in Supplementary Data) between April 1, 2002, to December 31, 2016. Identifying exposed individuals up to the end of 2016 ensured sufficient follow-up time after diagnosis to assess cost and health outcomes to December 31, 2019. We defined the index date as 5 days prior to the earlier of positive TB test result date recorded in the PHOL dataset or TB reporting date in iPHIS to account for the potential lag between testing date and diagnosis.

### Unexposed individuals (non-TB)

We identified individuals without TB during the same time frame from a random subset of 20% of the general population in Ontario (approximately 3.27 million people) for matching. Unexposed individuals were assigned a pseudo-index date at random.

We excluded individuals in both groups if they did not live in Ontario, did not have Ontario’s provincial health insurance at the time of TB diagnosis/index, had a date of last contact more than 3 years or 9 years prior to index (for people 65 years or older, or under 65 years, respectively), had invalid or missing data on sex, age, birth date, and were over 110 years old.

### Matching

We used a combination of hard and propensity score matching using nearest neighbour matching without replacement on selected baseline covariates. We matched each person with TB to two individuals without TB on index date (± 90 days), age (± 5 years), sex, time since immigration to Canada as reported in the Immigration, Refugees, and Citizenship Canada’s Permanent Resident Database (± 5 years; or not-immigrant), income quintile, resource utilization band (RUB) defined using Johns Hopkins Adjusted Clinical Group (ACG® system) case-mix system with a 2-year lookback window,^19^ and the logit of the propensity score using a caliper width of 0.2 standard deviations.^20^ The propensity score model included the four dimensions of the Ontario Marginalization Index measured at baseline: households and dwellings, material resources, age and labour force, and racialized and newcomer populations.^21^ Because health care use and cost increase prior to death,^22,23^ we rematched individuals with TB who died within the observation period to 2 unexposed individuals who died. We matched on death date (± 90 days), sex, time since immigration (± 5 years), income quintile, resource utilization band, and the logit of the propensity score (which included the four dimensions of the Ontario Marginalization Index). We assessed balance of both matched cohorts using standardized differences with a threshold of 0.10.^24^

### Outcomes

We calculated survival-adjusted health care costs from index date until death, censoring (i.e., lost provincial health insurance eligibility), or the end of the observation period (December 31, 2019) using the ICES person-level costing methodology.^25^ We ended the observation period at the end of 2019 because health service access was impacted by the COVID-19 pandemic. We report costs in 2020 Canadian dollars. We included the following publicly funded services: inpatient hospitalizations, emergency department visits, same-day surgeries, complex continuing care, physician services, diagnostic tests, eligible prescription medications, assistive devices, home care, and inpatient rehabilitation.^25^

### Analyses

**Figure 1.**
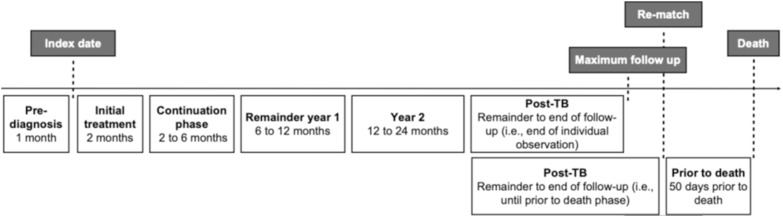
Phases of care for TB. The timeline depicts the phases of care that were defined for TB. The post-TB phase differs based on whether the individual died during the observation window; the post-TB phase lasted from the end of Year 2 until the end of the observation period for individuals who did not die (i.e., until December 31, 2019 or censoring). Individuals with TB who died during the observation window had a post-TB phase that lasted from the end of Year 2 until 50 days prior to death, at which time they were rematched and had a final phase lasting the 50 days prior to death. Figure is based off a prior phase-of-care costing study.^34^

We assessed health outcomes: 1-year and 5-year mortality, and acute hospital admissions (between 30 days prior to index and 30 days post index date). We assessed cost using a phase-based costing approach, which involves calculating the cost per phase of care standardized to a 10-day period. We determined phase of care length through a combination of joinpoint analysis, clinical expertise (SB, ER), and the Canadian TB diagnosis and treatment guidelines.^26^ For joinpoint regression analysis, we plotted mean daily costs from 1-year prior to index date to 1-year post-index date, and daily costs 1 year prior to death to identify the best-fitting points where statistically significant changes occurred. We defined seven phases of care: pre-diagnosis (1 month prior to index), initial treatment phase (2 months post-index), continuation phase (2 to 6 months post-index), remainder year 1 (6 to 12 months post-index), year 2 (12 to 24 months post-index), prior to death (50 days prior to death), and post-TB (time between the end of year 2 and the end of the observation period or the prior-to-death phase for decedents) (Figure 1). We used generalized estimating equations to calculate the mean difference in costs (costs attributable to TB) between exposed and unexposed individuals for each phase of care treating matched sets as clusters, using a gamma distribution, an exchangeable correlation structure, and an identity link.^27,28^ We conducted stratified analyses on income quintile and immigration status. We estimated survival-adjusted attributable mean 1-, 2-, and 5-year costs by applying 10-day survival probabilities to the mean 10-day phase costs.^29^ All analyses were performed using SAS 9.4 (SAS Institute, Cary, NC).

## RESULTS

Of 8,636 individuals with TB index dates between April 1, 2002 to December 31, 2016, 7,913 individuals (Supplementary Data Table S3) met cohort inclusion criteria. The cohort of individuals with TB had a mean (SD) age of 48.22 (21.59) years and 47.4% were female. Among the 2,009,504 unexposed individuals, the mean (SD) age was 38.78 (22.31) years, and 50.8% were females. In the unmatched cohorts, individuals with TB were more likely to be in the lowest income quintile compared to unexposed individuals and were more likely to be immigrants or refugees (62.4% of exposed individuals, 14.9% of unexposed individuals).

We matched 6,456 individuals with TB to 12,443 individuals without TB (Table 1). Following matching, the mean age of exposed and unexposed individuals was 47.04 and 46.35 years, respectively, and in both cohorts approximately 48.5% were female. Following matching, most individuals were in the two lowest income quintiles (∼60%), nearly half were recent residents (∼48%), and most lived in urban areas (98%). Baseline characteristics of the matched and unmatched cohort are presented in Table 1. The matched cohort was well balanced with all standardized differences less than 0.10. Among individuals with TB who died, 1,696 were re-matched. The mean (SD) age at death for the re-matched individuals with TB was 70.7 years, and 63.7% were males. Of individuals with TB who died, 32.7% were classified as recent residents, and 60.8% as born in Canada or immigrated prior to 1985; 5.5% (n = 93) were classified as refugees. All weighted standardized differences were less than 0.10 indicating a well-balanced match (Supplementary Data Table S4).

**Table 1.** Baseline characteristics of individuals exposed and unexposed to tuberculosis, prior to and after matching at index date.

Variable	Before matching		After matching		
	Exposed (n = 7,913)	Unexposed (n = 2,009,504)	Exposed (n = 6,456)	Unexposed (n = 12,443)	Weighted standardized differences
*Age*					
Mean (SD)	48.22 (21.59)	38.78 (22.31)	47.04 (20.87)	46.35 (20.26)	0.03
Median (Q1-Q3)	46 (31-66)	39 (20-55)	44 (31-64)	44 (31-62)	0.02
*Sex*					
Female	3,751 (47.4%)	1,020,507 (50.8%)	3,123 (48.4%)	6,033 (48.5%)	0.00
Male	4,162 (52.6%)	988,997 (49.2%)	3,333 (51.6%)	6,410 (51.5%)	0.00
*Neighbourhood income quintile*					
Missing data	35 (0.4%)	7,198 (0.4%)	—	—	—
1 (lowest)	2,913 (36.8%)	393,329 (19.6%)	2,407 (37.3%)	4,689 (37.7%)	0.01
2	1,802 (22.8%)	396,056 (19.7%)	1,498 (23.2%)	2,901 (23.3%)	0.00
3	1,411 (17.8%)	399,784 (19.9%)	1,139 (17.6%)	2,176 (17.5%)	0.00
4	1,060 (13.4%)	409,322 (20.4%)	863 (13.4%)	1,634 (13.1%)	0.01
5 (highest)	692 (8.7%)	403,815 (20.1%)	549 (8.5%)	1,043 (8.4%)	0.00
*Immigration status*					
Recent resident	3,920 (49.5%)	250,213 (12.5%)	3,082 (47.7%)	6,005 (48.3%)	0.01
Long-time resident	2,894 (36.6%)	1,705,545 (84.9%)	2,528 (39.2%)	5,008 (40.2%)	0.02
Refugee	1,020 (12.9%)	49,121 (2.4%)	798 (12.4%)	1,293 (10.4%)	0.06
Missing	79 (1.0%)	4,623 (0.2%)	48 (0.7%)	137 (1.1%)	0.04
*Resource utilization band*					
0 (non-utilizer)	634 (8.0%)	261,854 (13.0%)	379 (5.9%)	732 (5.9%)	0.00
1	307 (3.9%)	144,213 (7.2%)	229 (3.5%)	431 (3.5%)	0.01
2	947 (12.0%)	418,747 (20.8%)	763 (11.8%)	1,481 (11.9%)	0.00
3	3,395 (42.9%)	889,376 (44.3%)	3,072 (47.6%)	6,040 (48.5%)	0.02
4	1,514 (19.1%)	224,133 (11.2%)	1,279 (19.8%)	2,438 (19.6%)	0.01
5 (high complexity)	1,116 (14.1%)	71,181 (3.5%)	734 (11.4%)	1,321 (10.6%)	0.02
*Rurality*					
Rural	95 (1.2%)	150,075 (7.5%)	82 (1.3%)	196 (1.6%)	0.03
Urban	7,508 (97.6%)	1,833,832 (91.3%)	6,352 (98.4%)	12,212 (98.1%)	0.04
Missing Data	90 (1.2%)	25,597 (1.3%)	22 (0.3%)	35 (0.3%)	0.01
*Ontario Marginalization Index*					
*Age and labour force*					
1 (least marginalized)	2,604 (32.9%)	497,272 (24.7%)	2,196 (34.0%)	4,186 (33.6%)	0.01
2	1,966 (24.8%)	414,073 (20.6%)	1,651 (25.6%)	3,181 (25.6%)	0.00
3	1,302 (16.5%)	370,632 (18.4%)	1,088 (16.9%)	2,133 (17.1%)	0.01
4	952 (12.0%)	347,866 (17.3%)	775 (12.0%)	1,588 (12.8%)	0.02
5 (most marginalized)	962 (12.2%)	355,803 (17.7%)	746 (11.6%)	1,355 (10.9%)	0.02
Missing data	127 (1.6%)	23,858 (1.2%)	—	—	—
*Material resources*					
1 (least marginalized)	811 (10.2%)	403,650 (20.1%)	660 (10.2%)	1,199 (9.6%)	0.02
2	974 (12.3%)	395,999 (19.7%)	808 (12.5%)	1,553 (12.5%)	0.00
3	1,336 (16.9%)	385,596 (19.2%)	1,114 (17.3%)	2,068 (16.6%)	0.02
4	1,701 (21.5%)	386,599 (19.2%)	1,382 (21.4%)	2,829 (22.7%)	0.03
5 (most marginalized)	2,964 (37.5%)	413,802 (20.6%)	2,492 (38.6%)	4,794 (38.5%)	0.00
Missing data	127 (1.6%)	23,858 (1.2%)	—	—	—
*Racialized and newcomer populations*					
1 (least marginalized)	256 (3.2%)	323,030 (16.1%)	212 (3.3%)	406 (3.3%)	0.00
2	298 (3.8%)	340,033 (16.9%)	226 (3.5%)	484 (3.9%)	0.02
3	558 (7.1%)	361,959 (18.0%)	433 (6.7%)	804 (6.5%)	0.01
4	1,449 (18.3%)	410,101 (20.4%)	1,157 (17.9%)	2,172 (17.5%)	0.01
5 (most marginalized)	5,225 (66.0%)	550,523 (27.4%)	4,428 (68.6%)	8,577 (68.9%)	0.01
Missing Data	127 (1.6%)	23,858 (1.2%)	—	—	—
*Households and dwellings*					
1 (least marginalized)	1,927 (24.4%)	429,479 (21.4%)	1,615 (25.0%)	2,923 (23.5%)	0.04
2	971 (12.3%)	386,588 (19.2%)	786 (12.2%)	1,468 (11.8%)	0.01
3	954 (12.1%)	367,606 (18.3%)	806 (12.5%)	1,548 (12.4%)	0.00
4	1,369 (17.3%)	375,426 (18.7%)	1,127 (17.5%)	2,419 (19.4%)	0.05
5 (most marginalized)	2,565 (32.4%)	426,547 (21.2%)	2,122 (32.9%)	4,085 (32.8%)	0.00
Missing data	127 (1.6%)	23,858 (1.2%)	—	—	—

Long-time resident is defined as being born in Canada (including Indigenous Canadians) or immigrating to Canada prior to 1985. Recent residents include permanent residents who immigrated to Canada after 1985. The following information pertains to the components of the Ontario Marginalization Index; Households and dwellings (previously called ‘Residential instability’): Includes indicators that measure types and density of residential accommodations, and certain family structure characteristics, such as % living alone and % dwellings not owned; Material resources (previously called ‘Material deprivation’): Includes indicators that measure access to and attainment of basic material needs, such as % unemployment and % without a high school degree; Age and labour force (previously called ‘Dependency’): Includes indicators to describe % seniors (65+), the dependency ratio (the ratio of seniors and children to the population 15-64) and % not participating in the labour force; Racialized and newcomer populations (previously called ‘Ethnic concentration’): Includes indicators to describe % recent immigrants and % who self-identify as a ‘visible minority’ (as defined by Statistics Canada).^21^ Q1 = quartile one; Q3 = quartile three; SD = standard deviation.

### Health outcomes

Within 30 days prior to and 30 days following index date, 24% of individuals with TB were hospitalized with a mean (median) length of stay of 25.6 days (16 days). This finding differed by rurality: 24% in urban areas and 34% in rural areas were hospitalized, with mean (median) lengths of stay of 25.5 days (16 days) and 26.3 days (23 days), respectively. Within 1 year of index, 8% died (all-cause mortality) and 14% within 5 years of index date.

### TB-attributable health care costs

**Figure 2.**
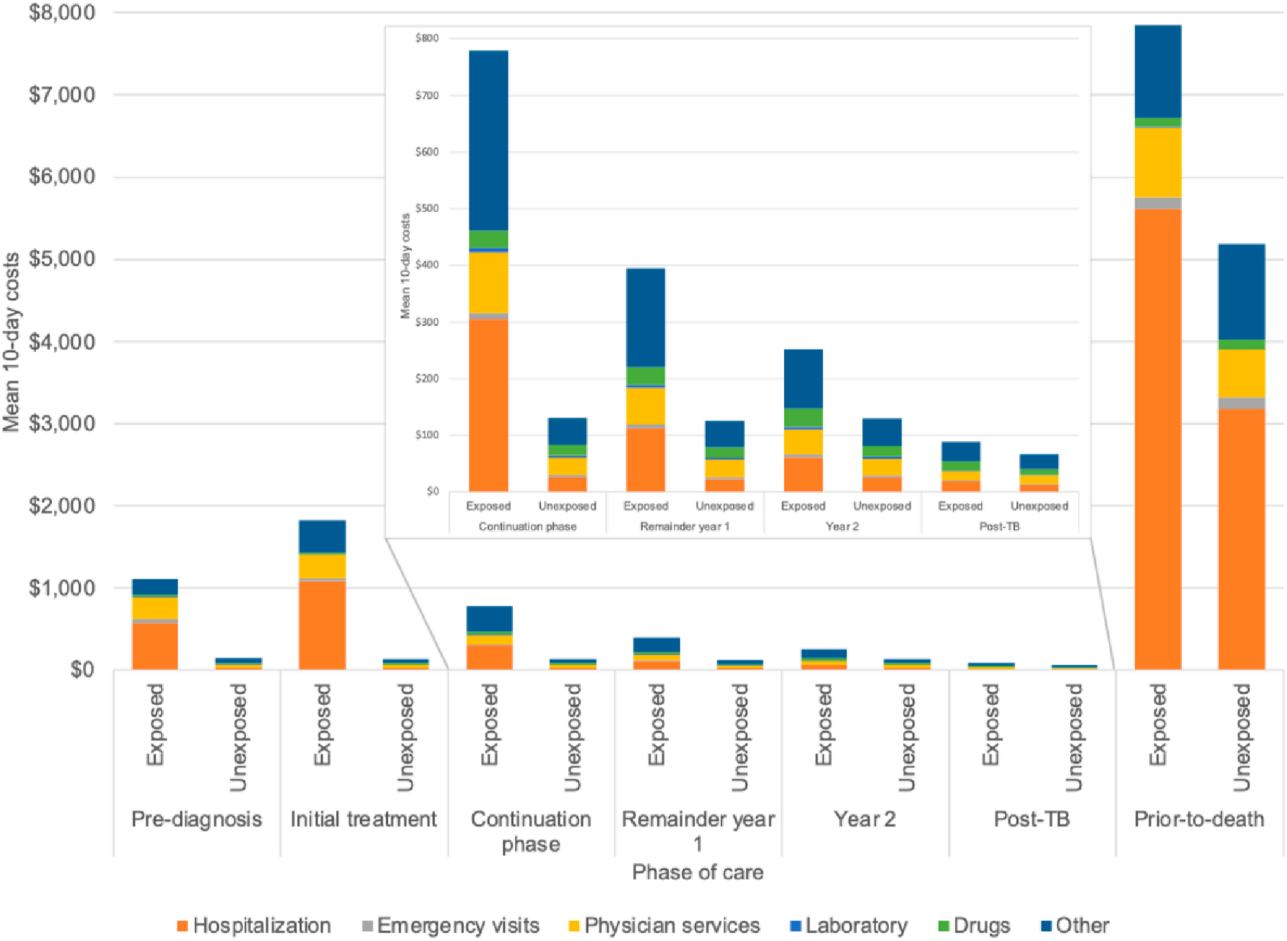
Mean 10-day costs (2020 Canadian dollars) for exposed and unexposed individuals, by cost category, in Ontario, Canada. Hospitalization costs include salaried physician services; physician services provided to inpatients are included in physician costs. ‘Other; costs include long-term care, continuing care, rehabilitation, mental health, dialysis, cancer, assistive devices, and home care. Drug costs do not include tuberculosis medications.

**Figure 3.**
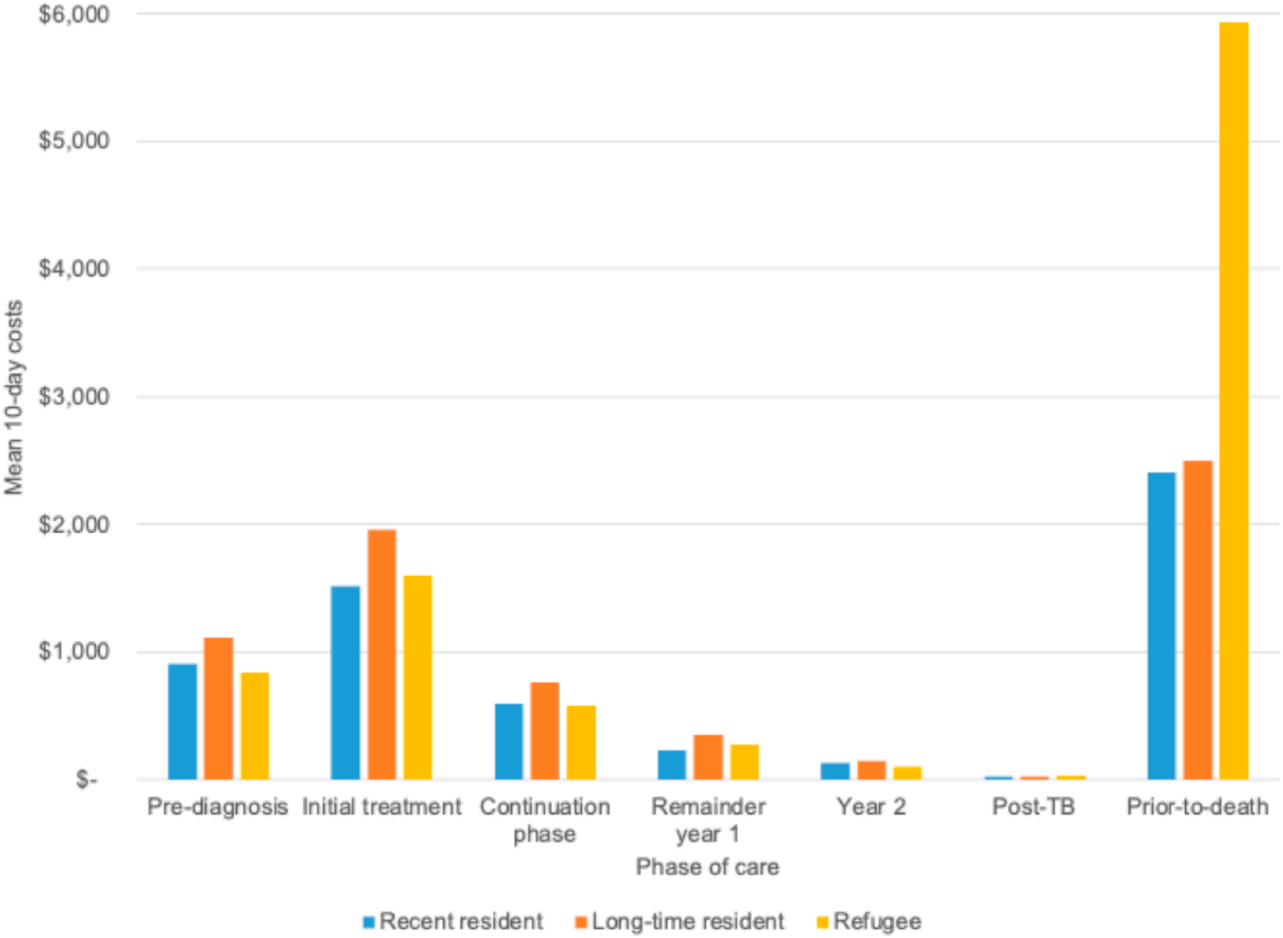
Mean 10-day attributable costs (2020 Canadian dollars) by phase of care stratified by immigration status for individuals with TB in Ontario, Canada.

The mean costs of people with TB are shown in Figure 2, with additional details in Supplementary Data Table S5. Mean 10-day TB-attributable costs by phase are reported in Table 2. Mean (95% CI) attributable 10-day costs were highest in the pre-death phase at $2,656 ($2,207, $3,104) followed by the initial treatment phase at $1,693 ($1,608, $1,778). The pre-diagnosis cost was $973 ($912, $1,035). Following initial treatment, costs declined over time in the continuing treatment, remainder year 1, year 2, and post-TB phases with 10-day costs of $656 ($608, 703), $280 ($250, 309), $129 ($103, $155), and $25 ($18, $31), respectively. Hospitalization was the largest cost component in most phases: 56% of pre-diagnosis cost, 62% of initial treatment, and 91% of the prior-to-death phase. Attributable costs declined over time from the initial treatment phase to the post-TB phase, before increasing in the prior-to-death phase. Stratified analyses demonstrated that TB-attributable costs differed by immigration status (i.e., refugee, recent resident, or long-time resident; defined as born in Canada or immigrated prior to 1985); with higher attributable costs in the pre-diagnosis and in phases up to the end of year 2 among long-time residents (Figure 3). Prior to death, the attributable costs were highest for individuals categorized as refugees; however, this group is notably smaller than other groups (93 exposed and 184 unexposed individuals). For attributable costs of TB disease by income quintiles, the pre-diagnosis phase demonstrated higher attributable costs for individuals with higher income (quintiles 4 and 5) (Supplementary Data Figure S1). Prior to death, the TB-attributable health care costs were highest for individuals in the lowest income quintiles (i.e., quintiles 1 and 2). The mean TB-attributable 1-, 2-, and 5-year costs adjusted for survival were $25,586, $30,178, and $33,370, respectively.

**Table 2.** Mean (95% CI) 10-day attributable costs (2020 Canadian dollars) by phase of care and cost category for individuals with tuberculosis in Ontario, Canada.

Cost category	Pre-diagnosis	Initial treatment	Continuation phase	Remainder year 1	Year 2	Post-TB	Prior-to-death
Total	973 (912, 1,035)	1,693 (1,608, 1,778)	656 (608, 703)	280 (250, 309)	129 (103, 155)	25 (18, 31)	2,656 (2,207, 3,104)
Hospitalization	545 (498, 591)	1,054 (985, 1,123)	279 (244, 314)	90 (70, 109)	36 (16, 55)	6 (3, 8)	2,430 (2,018, 2,843)
Emergency visits	48 (45, 51)	37 (35, 38)	8 (7, 8)	3 (3, 4)	2 (1, 2)	0 (0, 0)	4 (-4, 12)
Physician services	223 (211, 236)	246 (228, 264)	77 (72, 82)	35 (32, 38)	15 (12, 17)	1 (1, 2)	266 (217, 316)
Laboratory	7 (7, 8)	7 (6, 7)	4 (3, 4)	2 (1, 2)	1 (0, 1)	0 (0, 0)	-1 (-2, 0)
Drugs	8 (4, 11)	7 (4, 9)	12 (9, 15)	13 (10, 16)	13 (10, 16)	6 (5, 8)	-3 (-19, 14)
Other	142 (128, 156)	342 (320, 363)	269 (249, 290)	128 (112, 144)	57 (44, 70)	9 (4, 13)	-40 (-129, 49)

Hospitalization costs include salaried physician services; additional physician services provided to inpatients are included in physician costs. ‘Other’ costs include long-term care, continuing care, rehabilitation, mental health, dialysis, outpatient cancer clinics, assistive devices, and home care. Drug costs do not include TB medications. CI = confidence interval.

## DISCUSSION

TB resulted in increased health care costs from 30 days prior to index date until death. Attributable costs were highest in the first 2 months after diagnosis and prior to death, primarily due to hospitalization. Notably, TB attributable health care costs persisted over the long term in the post-TB phase indicating ongoing costs related to post-TB sequelae. This finding aligns with the literature on post-TB lung disease as a driver of long-term health care use,^6^ and warrants future research considering individual level and geographic variations. Approximately 24% of patients were hospitalized within 30 days of diagnosis, with an average stay of 26 days. In Ontario, hospital admissions are generally for clinical care rather than isolation alone.

Notably, in the stratified analyses, refugees (vs. long-time and recent residents) and individuals in the lowest income quintile (vs. higher income quintiles) had higher attributable costs in the prior-to-death phase. Higher costs among refugees may be partly due to their socioeconomic status, with 60% in the lowest income quintile compared to 30-35% among long-time and recent residents, respectively. Prior research in Canada has demonstrated that people in the lowest income group make up a higher proportion of direct health care costs than higher income groups.^30^ Health care costs of TB were estimated in a prior study at three TB centres in Canada using a retrospective chart review.^9^ While it is challenging to directly compare the results due to different methodologies (including cost categories and sampling), Campbell et al. similarly found that most costs associated with TB disease were associated with hospitalization. Similarly, a review of TB costs in European Union countries reported that hospitalizations accounted for a substantial portion of total direct health care expenditures, and differences in costs across countries.^13^

Our study is subject to limitations inherent in the design and use of health administrative data including misclassification and information bias.^31,32^ It is possible that individuals who received tests are more likely to access timely health care, thus excluding those without positive test results may impact the estimated cost of TB. However, TB is a mandatory reportable disease in Ontario for both physicians and laboratories; together with a standard provincial case definition this ensures consistent diagnosis and surveillance data. Another source of potential underreporting is the exclusion of individuals without provincial health insurance at the time of diagnosis. Excluding 4.7% of individuals without provincial health insurance (e.g., visitors, people without status, students, and some refugees) may have resulted in over- or underestimating health care costs depending on care-seeking behaviour and disease severity. However, most individuals in Ontario are covered by provincial public health insurance (an estimated 10% of TB occurs in temporary residents).^33^ Furthermore, the costs of TB are underreported as TB medication and public health TB costs are paid for by public health in Ontario and are not captured in the datasets we used. Finally, our study did not include data on indigenous identifiers so we cannot specifically estimate costs in this population compared to other populations.

Our study has important strengths. The use of Ontario's rich administrative data allowed us to analyze a broad spectrum of cost categories longitudinally, providing a comprehensive picture of the economic burden attributable to TB across all phases of care. The data’s large sample size, coupled with long-term follow-up spanning up to 17 years, ensures a robust assessment of costs throughout different stages of TB care. Moreover, integrating data from the IRCC Permanent Resident Database allowed for the assessment of costs stratified by important demographic characteristics. The combination of hard-matching and propensity score matching on key covariates further strengthens the internal validity of our cost estimates by reducing bias and confounding.

From a policy perspective, understanding the long-term costs of TB aids resource allocation and health care planning. By capturing both short- and long-term costs and stratifying the results by important socio-demographic characteristics, policymakers can better understand any differential impact that TB has on health spending across populations.

## Supplementary Material


