# Supplementary material for: A longitudinal analysis of the economic cost of all phases of TB care in a low incidence setting
Source: IJTLD Open. 2025 Jul 9;2(7):404–11. doi: 10.5588/ijtldopen.25.0076 (PMC12248407; doi:10.5588/ijtldopen.25.0076)
Supplement: Supplementary file 1 [file ijtldopen25-0076_supplementarydata1.pdf]

Supplemental Materials

Health care costs attributable to tuberculosis disease in Ontario, Canada: A population-based cohort study

Contents

1. Supplemental Table 1. Record Checklist
2. Supplemental Table 2. Description of ICES datasets
3. Ontario tuberculosis case definition
4. Supplemental Table 3. Cohort creation for exposed and unexposed individuals, prior to matching
5. Supplemental Table 4. Baseline characteristics of exposed and unexposed decedents, after matching
6. Supplemental Table 5: Mean 10-day standardized health care costs for exposed and unexposed individuals
7. Supplemental Figure 1: Mean 10-day attributable costs by phase of care stratified by income quintile for people with tuberculosis disease in Ontario, Canada

Supplemental Table 1. RECORD Checklist

Reference: Benchimol EI, Smeeth L, Guttmann A, Harron K, Moher D, Petersen I, Sørensen HT, von Elm E, Langan SM, the RECORD Working Committee. The REporting of studies Conducted using Observational Routinely-collected health Data (RECORD) Statement. *PLoS Medicine* 2015.

	Item No.	STROBE items		RECORD items	Reported on
**Title and abstract**					
	1	(a) Indicate the study's design with a commonly used term in the title or the abstract (b) Provide in the abstract an informative and balanced summary of what was done and what was found		RECORD 1.1: The type of data used should be specified in the title or abstract. When possible, the name of the databases used should be included.<br><br>RECORD 1.2: If applicable, the geographic region and timeframe within which the study took place should be reported in the title or abstract.<br><br>RECORD 1.3: If linkage between databases was conducted for the study, this should be clearly stated in the title or abstract.	P.1-2<br><br>P.1-2<br><br>P.1-2
**Introduction**					
Background rationale	2	Explain the scientific background and rationale for the investigation being reported			P.3
Objectives	3	State specific objectives, including any prespecified hypotheses			P.3
**Methods**					
Study Design	4	Present key elements of study design early in the paper			P.4
Setting	5	Describe the setting, locations, and relevant dates, including periods of recruitment, exposure, follow-up, and data collection			P.4
Participants	6	*(a) Cohort study* - Give the eligibility criteria, and the sources and methods of selection of participants. Describe methods of follow-up *Case-control study* - Give the eligibility criteria, and the sources and methods of case ascertainment and control selection. Give the rationale for the choice of cases and controls		RECORD 6.1: The methods of study population selection (such as codes or algorithms used to identify subjects) should be listed in detail. If this is not possible, an explanation should be provided.<br><br>RECORD 6.2: Any validation studies of the codes or algorithms used to select the population should be referenced. If validation was conducted for this study	P.4 Supplementary Materials<br><br>N/A

		Cross-sectional study - Give the eligibility criteria, and the sources and methods of selection of participants<br><br>*(b) Cohort study* - For matched studies, give matching criteria and number of exposed and unexposed<br>*Case-control study* - For matched studies, give matching criteria and the number of controls per case		and not published elsewhere, detailed methods and results should be provided.<br><br>RECORD 6.3: If the study involved linkage of databases, consider use of a flow diagram or other graphical display to demonstrate the data linkage process, including the number of individuals with linked data at each stage.	N/A
Variables	7	Clearly define all outcomes, exposures, predictors, potential confounders, and effect modifiers. Give diagnostic criteria, if applicable.		RECORD 7.1: A complete list of codes and algorithms used to classify exposures, outcomes, confounders, and effect modifiers should be provided. If these cannot be reported, an explanation should be provided.	P.6<br><br>Supplementary materials
Data sources/ measurement	8	For each variable of interest, give sources of data and details of methods of assessment (measurement).<br>Describe comparability of assessment methods if there is more than one group			P.5-6<br><br>Supplementary Materials
Bias	9	Describe any efforts to address potential sources of bias			P.5-6
Study size	10	Explain how the study size was arrived at			Supplementary Material
Quantitative variables	11	Explain how quantitative variables were handled in the analyses. If applicable, describe which groupings were chosen, and why			P.5-6
Statistical methods	12	(a) Describe all statistical methods, including those used to control for confounding<br>(b) Describe any methods used to examine subgroups and interactions<br>(c) Explain how missing data were addressed<br>(d) *Cohort study* - If applicable, explain how loss to follow-up was addressed<br>*Case-control study* - If applicable, explain how matching of cases and controls was addressed<br>*Cross-sectional study* - If applicable, describe analytical methods taking account of sampling strategy<br>(e) Describe any sensitivity analyses			P.5-6

Data access and cleaning methods		..		RECORD 12.1: Authors should describe the extent to which the investigators had access to the database population used to create the study population.<br><br>RECORD 12.2: Authors should provide information on the data cleaning methods used in the study.	P.10-11<br><br>Supplementary materials
Linkage		..		RECORD 12.3: State whether the study included person-level, institutional-level, or other data linkage across two or more databases. The methods of linkage and methods of linkage quality evaluation should be provided.	P.5-6
**Results**					
Participants	13	(a) Report the numbers of individuals at each stage of the study (*e.g.*, numbers potentially eligible, examined for eligibility, confirmed eligible, included in the study, completing follow-up, and analysed)<br>(b) Give reasons for non-participation at each stage.<br>(c) Consider use of a flow diagram		RECORD 13.1: Describe in detail the selection of the persons included in the study (*i.e.,* study population selection) including filtering based on data quality, data availability and linkage. The selection of included persons can be described in the text and/or by means of the study flow diagram.	P.7
Descriptive data	14	(a) Give characteristics of study participants (*e.g.*, demographic, clinical, social) and information on exposures and potential confounders<br>(b) Indicate the number of participants with missing data for each variable of interest<br>(c) *Cohort study* - summarise follow-up time (*e.g.*, average and total amount)			P.7-8
Outcome data	15	*Cohort study* - Report numbers of outcome events or summary measures over time<br>*Case-control study* - Report numbers in each exposure category, or summary measures of exposure<br>*Cross-sectional study* - Report numbers of outcome events or summary measures			P.7-8<br>Supplementary Materials
Main results	16	(a) Give unadjusted estimates and, if applicable, confounder-adjusted estimates and their precision (e.g., 95% confidence interval). Make clear which confounders were adjusted for and why they were included			P.7-8

		(b) Report category boundaries when continuous variables were categorized<br>(c) If relevant, consider translating estimates of relative risk into absolute risk for a meaningful time period			
Other analyses	17	Report other analyses done—e.g., analyses of subgroups and interactions, and sensitivity analyses			P.8
**Discussion**					
Key results	18	Summarise key results with reference to study objectives			P.8-9
Limitations	19	Discuss limitations of the study, taking into account sources of potential bias or imprecision. Discuss both direction and magnitude of any potential bias		RECORD 19.1: Discuss the implications of using data that were not created or collected to answer the specific research question(s). Include discussion of misclassification bias, unmeasured confounding, missing data, and changing eligibility over time, as they pertain to the study being reported.	P.9
Interpretation	20	Give a cautious overall interpretation of results considering objectives, limitations, multiplicity of analyses, results from similar studies, and other relevant evidence			P.8-9
Generalisability	21	Discuss the generalisability (external validity) of the study results			P.10
**Other Information**					
Funding	22	Give the source of funding and the role of the funders for the present study and, if applicable, for the original study on which the present article is based			P.11
Accessibility of protocol, raw data, and programming code		..		RECORD 22.1: Authors should provide information on how to access any supplemental information such as the study protocol, raw data, or programming code.	P.11

*Checklist is protected under Creative Commons Attribution (CC BY) license.

Supplemental Table 1. Descriptions of ICES datasets
Reference: *ICES Data Dictionary*. Retrieved October 25, 2024, from https://datadictionary.ices.on.ca/Applications/DataDictionary/Default.aspx

**Continuing Care Reporting System (CCRS)**
The CCRS database is compiled by the Canadian Institute for Health Information and contains demographic, clinical, functional, and resource utilization information for individuals receiving facility-based continuing care (also known as extended, auxiliary, or complex chronic care) in Ontario hospitals and residential care providing 24 hour nursing services (i.e. nursing home). Clinical assessment data (on the physical, functional, cognitive, and social domains of health) is ascertained using the Resident Assessment Instrument Minimum Data Set (RAI-MDS) version 2.0 which is administered by trained healthcare professionals.
**Discharge Abstract Database (CIHI-DAD)**
The DAD is compiled by the Canadian Institute for Health Information and contains administrative, clinical (diagnoses and procedures/interventions), demographic, and administrative information for all admissions to acute care hospitals, rehab, chronic, and day surgery institutions in Ontario. At ICES, consecutive DAD records are linked together to form ‘episodes of care’ among the hospitals to which patients have been transferred after their initial admission.
**National Ambulatory Care Reporting System (NACRS)**
The NACRS is compiled by the Canadian Institute for Health Information and contains administrative, clinical (diagnoses and procedures), demographic, and administrative information for all patient visits made to hospital- and community-based ambulatory care centres (emergency departments, day surgery units, hemodialysis units, and cancer care clinics). At ICES, NACRS records are linked with other data sources (DAD, OMHRS) to identify transitions to other care settings, such as inpatient acute care or psychiatric care.
**National Rehabilitation Reporting System (NRS)**
The NRS is compiled by the Canadian Institute for Health Information and contains client data collected from participating adult inpatient rehabilitation facilities and programs across Canada. Main data elements contain socio-demographic information, administrative data (e.g. referral, admission and discharge), health characteristics, activities and participation (e.g. ADL, communication, social interaction), and interventions.
**Ontario Drug Benefit Claims (ODB)**
The ODB database contains prescription medication claims for those covered under the provincial drug program, mainly: those aged 65 years and older, nursing home residents, patients receiving services under the Ontario Home Care program, those receiving social assistance, and residents eligible for specialized drug programs. Main data elements include drug identifier, quantity, # days supplied, date dispensed, cost, and patient, pharmacy and physician identifiers.
**Ontario Health Insurance Plan (OHIP)**
The OHIP claims database contains information on inpatient and outpatient services provided to Ontario residents eligible for the province’s publicly funded health insurance system by fee-for-service health care practitioners (primarily physicians) and “shadow billings” for those paid through non-fee-for-service payment plans. The main data elements include patient and physician identifiers (encrypted), code for service provided, date of service, associated diagnosis, and fee paid.
**Same-Day Surgery (SDS)**

The SDS is compiled by the Canadian Institute for Health Information and contains administrative, clinical (diagnoses and procedures), demographic, and administrative information for all patient visits made to day surgery institutions in Ontario. The main data elements include patient demographics, clinical data (diagnoses, procedures, physician), administrative data (institution/hospital number etc.), financial data, service-specific data elements for day surgery and emergency.
**Assistive Devices Program (ADP)**
The Assistive Devices Program (ADP) provides consumer-centered support and funding to Ontario residents who have long-term physical disabilities, and to provide access to personalized assistive devices appropriate for the individual's basic needs. The available data files include general information on select devices, renewal, insulin pumps & supplies, home oxygen, respiratory equipment & supplies, and ventilator equipment & supplies.
**Registered Persons Database (RPDB)**
The RPDB provides basic demographic information (age, sex, location of residence, date of birth, and date of death for deceased individuals) for those issued an Ontario health insurance number. The RPDB also indicates the time periods for which an individual was eligible to receive publicly funded health insurance benefits and the best known postal code for each registrant on July 1st of each year.
**Immigration, Refugees, and Citizenship Canada's (IRCC) Permanent Resident Database**
The Ontario portion of the IRCC Permanent Resident Database includes immigration application records for people who initially applied to land in Ontario since 1985. The dataset contains permanent residents' demographic information such as country of citizenship, level of education, mother tongue, and landing date. New immigrants who are currently residing in Ontario but originally landed in another province are not captured in this dataset.
**HIV**
The Ontario HIV Database is an ICES-derived cohort that is created using a definition of ≥3 physician billing claims with a diagnosis of HIV (OHIP diagnosis codes: 042, 043, 044) in a three-year period applied to physician billing claims (OHIP) data to determine the diagnosis date for incident cases of HIV in Ontario.
**Ontario Marginalization Index (ONMARG)**
ONMARG is a geographically (census) based index developed to quantify the degree of marginalization occurring across the province of Ontario. It is comprised of four major dimensions thought to underlie the construct of marginalization: household and dwellings, material resources, age and labour force, and racialized and newcomer populations. The dataset contains census divisions (CD), census tracts (CT), census subdivisions (CSD), consolidated municipal service manager areas (CMSM), public health units (PHU), local health integration networks (LHIN), sub-LHINs, and dissemination areas (DA).
**Home Care Database (HCD)**
The HCD is a clinical client centric database that captures all services that are provided by or coordinated by Community Care Access Centres (CCACs). The data elements captured include information on: client, intake, assessment, admission & discharge, diagnosis and surgical procedure, and care delivery. ICES receives home care data from the Ontario Ministry of Health and Long-Term Care (MOHLTC). The primary purpose of the information collected through the HCD is to aid in planning and better clinical insight into clients who encounter service through CCACs.
**Client Agency Program Enrolment (CAPE)**

The CAPE Database is a registry of all patients who have ever been rostered to receive care from a particular physician in Ontario and documents the time period in which a patient was rostered to a specific physician. A new record is created when a Registered Person enrolls in a program. The individual must be eligible for Ontario Health Insurance at the time of rostering.
**Ontario Mental Health Reporting System (OMHRS)**
The OMHRS is compiled by the Canadian Institute for Health Information and contains administrative, clinical (diagnoses and procedures), demographic, and administrative information for all admissions to adult designated inpatient mental health beds. This includes beds in general hospitals, provincial psychiatric facilities, and specialty psychiatric facilities. Clinical assessment data is ascertained using the Resident Assessment Instrument for Mental Health (RAI-MH), but different amounts of information are collected using this instrument depending on the length of stay in the mental health bed. Multiple assessments may occur during the length of a mental health admission.

Ontario Tuberculosis Case Definition

Reference: *Ontario Ministry of Health. (2022). Ontario Public Health Standards: Requirements for Programs, Services and Accountability Infectious Disease Protocol. Appendix 1: Case definitions and disease specific information. Disease: Tuberculosis. https://files.ontario.ca/moh-ophs-tuberculosis-en-2022.pdf*

Laboratory confirmed active TB will be defined following the Ontario Ministry of Health and Long Term Care (MOHLTC) Infectious Diseases Protocol:

- cases with *Mycobacterium tuberculosis* complex (MTB complex) demonstrated on culture from an appropriate clinical specimen (e.g., sputum, body fluid or tissue) specifically *M. tuberculosis*, *M. africanum*, *M. canetti*, *M. caprae*, *M. microti, M. pinnipedii or M. bovis* (excluding *M. bovis* Bacillus Calmette Guérin [BCG] strain)

Clinical TB will be defined following Ontario's MOHLTCs Infectious Diseases Protocol, Appendix B for TB [1]:

- In the absence of positive culture, cases clinically compatible with active tuberculosis that have:
    - Chest radiological changes compatible with active tuberculosis; OR
    - Histopathologic or post-mortem evidence of active tuberculosis; OR
    - Response to anti-tuberculosis treatment; OR
    - Detection of MTB complex by NAAT with compatible clinical and epidemiological associated information; OR
    - Active non-respiratory tuberculosis (meningeal, bone, kidney, peripheral lymph nodes, etc.).

Supplemental Table 3. Cohort creation, prior to matching

Description	Exposed Individuals			Unexposed Individuals		
	Included	Excluded		Included	Excluded	
	n	n	%	n	n	%
**Index dates between April 1 2002 and December 31 2016**	8,636	-	-	3,269,351	-	-
Not living in Ontario (at index date)	8,565	71	0.6	2,582,782	686,569	16.1
Death date before index date	8,453	112	1.0	2,575,822	6,960	0.2
Age > 110 at index date	8,453	0	0.0	2,575,631	191	0.0
DOLC < index date - 3 (age≥65) or DOLC < index date - 9 (age<65)	8,438	15	0.1	2,393,101	182,530	4.3
Not OHIP eligible (at index date)	7,913	525	4.7	2,009,523	383,578	9.0
**Study Cohort**	**7,913**			**2,009,523**		

Abbreviations: DOLC = date of last contact; OHIP = Ontario Health Insurance Plan

Supplemental Table 4. Baseline characteristics of exposed and unexposed decedents, after matching

Variable	Exposed (n = 1,696)	Unexposed (n =3,231)	Weighted Standardized Differences
**Age**			
Mean (SD)	70.70 (14.96)	69.19 (16.03)	0.097
Median (Q1-Q3)	74 (62-81)	72 (60-81)	0.088
**Sex**			
Female	616 (36.3%)	1,167 (36.1%)	0.004
Male	1,080 (63.7%)	2,064 (63.9%)	0.004
**Neighbourhood Income Quintile**			
1 (lowest)	570 (33.6%)	1,109 (34.3%)	0.015
2	405 (23.9%)	770 (23.8%)	0.001
3	316 (18.6%)	594 (18.4%)	0.006
4	227 (13.4%)	420 (13.0%)	0.011
5 (highest)	178 (10.5%)	338 (10.5%)	0.001
**Immigration Status**			
Recent resident	555 (32.7%)	957 (29.6%)	0.067
Long time resident	1,031 (60.8%)	2,056 (63.6%)	0.059
Refugee	93 (5.5%)	184 (5.7%)	0.009
Missing	17 (1.0%)	34 (1.1%)	0.005
**Resource Utilization Band**			
0 (non-utilizer)	74 (4.4%)	136 (4.2%)	0.008
1	16 (0.9%)	29 (0.9%)	0.005
2	65 (3.8%)	117 (3.6%)	0.011
3	618 (36.4%)	1,201 (37.2%)	0.015
4	376 (22.2%)	710 (22.0%)	0.005
5 (high complexity)	547 (32.3%)	1,038 (32.1%)	0.003
**Ontario Marginalization Index**			
**Age and labour force**			
1 (least marginalized)	422 (24.9%)	811 (25.1%)	0.005
2	390 (23.0%)	695 (21.5%)	0.036
3	298 (17.6%)	613 (19.0%)	0.036
4	245 (14.4%)	418 (12.9%)	0.044
5 (most marginalized)	341 (20.1%)	694 (21.5%)	0.034
**Material Resources**			
1 (least marginalized)	178 (10.5%)	323 (10.0%)	0.016
2	243 (14.3%)	429 (13.3%)	0.03
3	296 (17.5%)	542 (16.8%)	0.018
4	396 (23.3%)	797 (24.7%)	0.031

5 (most marginalized)	583 (34.4%)	1,140 (35.3%)	0.019
**Racialized and newcomer populations**			
1 (least marginalized)	108 (6.4%)	232 (7.2%)	0.032
2	103 (6.1%)	211 (6.5%)	0.019
3	143 (8.4%)	258 (8.0%)	0.016
4	333 (19.6%)	619 (19.2%)	0.012
5 (most marginalized)	1,009 (59.5%)	1,911 (59.1%)	0.007
**Households and dwellings**			
1 (least marginalized)	383 (22.6%)	729 (22.6%)	0.000
2	213 (12.6%)	419 (13.0%)	0.012
3	204 (12.0%)	414 (12.8%)	0.024
4	326 (19.2%)	566 (17.5%)	0.044
5 (most marginalized)	570 (33.6%)	1,103 (34.1%)	0.011

Abbreviations: Q1 = quartile one; Q3 = quartile three; SD = standard deviation.
Note: Long time resident is defined as being born in Canada or immigrating to Canada prior to 1985. The following information pertains to the components of the Ontario Marginalization Index; **Households and dwellings** (previously called 'Residential instability'): Includes indicators that measure types and density of residential accommodations, and certain family structure characteristics, such as % living alone and % dwellings not owned; **Material resources** (previously called 'Material deprivation'): Includes indicators that measure access to and attainment of basic material needs, such as % unemployment and % without a high school degree; **Age and labour force** (previously called 'Dependency'): Includes indicators to describe % seniors (65+), the dependency ratio (the ratio of seniors and children to the population 15-64) and % not participating in the labour force; **Racialized and newcomer populations** (previously called 'Ethnic concentration'): Includes indicators to describe % recent immigrants and % who self-identify as a 'visible minority' (as defined by Statistics Canada).
Reference: Matheson FI (Unity Health Toronto), Moloney G (Unity Health Toronto), van Ingen T (Public Health Ontario). 2021 Ontario marginalization index: user guide. Toronto, ON: St. Michael's Hospital (Unity Health Toronto); 2023. Joint publication with Public Health Ontario. https://www.publichealthontario.ca/-/media/Documents/O/2017/on-marg userguide.pdf?rev=06cc3a5e23d4448ab6851b528756c428&sc_lang=en&hash=6E2098165339B008502D273C397BC699

Supplemental Table 5. Mean 10-day standardized health care costs for individuals exposed and unexposed to tuberculosis, 2020 Canadian dollars

Phase of care	Cost Category	Exposed	Unexposed
**Pre-diagnosis**	N	6,456	12,443
	Total	1,114.10	140.44
	Hospitalization	573.95	29.09
	Emergency Visits	51.77	3.6
	Physician Services	256.35	32.81
	Laboratory	11.34	3.99
	Drugs	25.88	18.24
	Other	194.84	52.75
**Initial Treatment**	N	6,441	12,441
	Total	1,826.44	133.21
	Hospitalization	1,083.29	28.84
	Emergency Visits	40.24	3.73
	Physician Services	277.03	30.98
	Laboratory	10.73	3.87
	Drugs	24.69	18.07
	Other	389.59	47.75
**Continuation Phase**	N	6,354	12,421
	Total	784.03	130.98
	Hospitalization	305.08	26.4
	Emergency Visits	11.15	3.59
	Physician Services	107.06	30.48
	Laboratory	7.66	3.95
	Drugs	30.61	18.64
	Other	317.41	47.87
**Remainder Year 1**	N	6,244	12,371
	Total	400.65	125.16
	Hospitalization	112.63	23.26
	Emergency Visits	6.93	3.65
	Physician Services	64.12	29.69
	Laboratory	5.65	3.99
	Drugs	31.21	18.25
	Other	174.23	46.09
**Year 2**	N	6,151	12,270
	Total	257.02	130.23
	Hospitalization	61.13	25.37

Phase of care	Cost Category	Exposed	Unexposed
	Emergency Visits	5.33	3.71
	Physician Services	44.28	29.82
	Laboratory	4.4	3.83
	Drugs	31.69	18.99
	Other	105.01	47.87
**Post-TB**	N	5,998	12,105
	Total	91.1	67.07
	Hospitalization	18.33	12.66
	Emergency Visits	2.15	1.97
	Physician Services	15.69	14.29
	Laboratory	1.68	1.68
	Drugs	16.04	9.97
	Other	34.58	25.79
**Prior-to-death**	N	1,696	3,231
	Total	7,847.41	5,188.14
	Hospitalization	5,614.05	3,181.77
	Emergency Visits	141.97	137.68
	Physician Services	847.54	580.78
	Laboratory	6.71	7.4
	Drugs	114.24	117.13
	Other	1,122.92	1,163.40

Notes: Hospitalization costs include salaried physician services; physician services provided to inpatients are included in physician costs. Other costs include long-term care, continuing care, rehabilitation, mental health, dialysis, cancer, assistive devices, and home care.

Supplemental Figure 1. Mean 10-day attributable costs (2020 Canadian dollars) by phase of care stratified by income quintile for people with tuberculosis in Ontario, Canada

Abbreviations: TB = tuberculosis
